# Transitions to adulthood among U.S. young adults with mental health disabilities before and during the COVID-19 pandemic: traveling multiple pathways in uncertain times

**DOI:** 10.3389/fpsyt.2026.1911182

**Published:** 2026-09-11

**Authors:** Judith A. Cook, Frances Aranda, Jessica A. Jonikas, Melania Boker, Claudia Cortez, DeVon Richardson, Beth Bendtsen, Alexandra E. Julian, Kathryn Sabella

**Affiliations:** 1 Department of Psychiatry, University of Illinois Chicago, Chicago, IL, United States; 2 Department of Psychiatry and Behavioral Sciences, UMass Chan Medical School, Worcester, MA, United States

**Keywords:** COVID-19 pandemic, disabling mental illness, life transitions, pandemic transition disruption, transition age young adult, transition to adulthood, young adult, youth with psychiatric disability

## Abstract

**Purpose:**

The impact of the COVID-19 pandemic on young adults with serious mental illnesses is poorly understood despite the high likelihood that important milestones for this group were affected by the pandemic and its economic aftermath. This study explored how U.S. young adults with disability due to mental health disorders experienced transitions to adulthood before and during the pandemic.

**Methods:**

Semi-structured qualitative interviews with 20 emerging adults, age 18-25 (mean age, 22.3) including 10 female, 4 male, and 6 gender non-binary or other gender individuals, covered 7 life transitions identified from the literature: residential independence, school completion, friendship network development, individuation from family, full-time employment, marriage/intimate partner establishment, and parenthood. Summative content analysis and inductive thematic analysis were conducted by a team including young adult co-researchers with lived experience of mental health challenges and disabilities.

**Results:**

A substantial number of transitions completed before the pandemic included graduation, residential independence, forming friendship networks, and individuation from family. Lockdown often derailed transitions, especially education and employment; and led to abandonment or postponement of other transitions, especially romantic relationships and full-time work. Educational interruptions reverberated beyond academics to include food insecurity, economic instability, and residential uncertainty. Life course concerns included not meeting cultural expectations of timely transitions, and distress over the disconnect between personal aspirations and current circumstances. Resilience was also observed, especially concerning mutual assistance from social networks and feelings of pride at being able to successfully negotiate pandemic stressors.

**Conclusion:**

Despite significant psychiatric multi-morbidity and pandemic-related disruptions, participants demonstrated a largely normative pre-pandemic life trajectory and showed notable resilience in the face of adversity. Findings suggest the need for transition-focused wraparound service systems, early intervention strategies to avoid development of further comorbidities, and supports incorporating informal social networks such as friends and family.

## Introduction

Almost a tenth (9.7%) of U.S. young people age 18 to 25 experience serious mental illness (1), defined as a mental health condition accompanied by moderate to severe impairment in one or more aspects of daily living (2). This often leads to delays in cognitive, social, and emotional development that negatively impact transitions to adulthood such as school completion, residential independence, parenthood, and launching a career (3). Studies show that young people’s mental health is both a cause and consequence of disrupted transitions (4, 5), yet little research has addressed the impact of the COVID-19 pandemic and its aftermath on young people with disabling mental health conditions (6). This study involved an in-depth exploration of the transitions to adulthood of a group of U.S. community-dwelling young adults with disabling mental health conditions, particularly their transition experiences before and during the pandemic, and what these reveal about their resilience and needs for further services and supports.

Young adult life transitions examined in prior research include completion of high school or post-secondary education (7), obtaining stable full-time employment (8), marriage or establishment of an intimate partner relationship (9), attainment of parenthood (10), achieving residential independence (11), separation-individuation from family (12), and creation of a satisfying social network (13). While considerable research documents the pandemic’s negative effects on the mental health of U.S. youth and young adults (14–17), fewer studies focus specifically on transition experiences in the general population. In a cohort of Irish young adults in their early twenties surveyed pre- and post-pandemic (18), the large majority reported pandemic-related interruptions of their work, education, and social activities. Research on U.S. young adults who experienced discomfort when residing involuntarily with parents during the pandemic (19, 20) revealed that mental health declines were associated with low autonomy, poorer parent–child interactions, and less positive coping. Studies of young adult job loss during the pandemic found associations between unemployment and poorer psychological well-being (21, 22). Finally, a qualitative study of close relationships during the pandemic reported by 707 U.S. college students found that negative changes in romantic relationships were associated with both physical and emotional distancing due to isolation and loneliness (23).

We located only one study of transition interruptions among young adults with self-reported psychiatric disability, involving a survey conducted in March through June of 2021 (24). Results revealed that 81% reported one or more disrupted transitions including delays in education completion (38%), securing jobs in chosen career areas (37%), establishing residential independence (27%), and forming intimate relationships (22%). In multivariable analyses, disruption in establishing intimate partner relationships was associated with depression, anxiety, and trauma symptoms, interrupted residential independence was associated with anxiety, interrupted education completion with symptoms of trauma, and interrupted employment with anxiety (ibid.). Social determinants also significant in these models included social connections, community participation, income, and racial/ethnic identification (ibid.).

The U.S. pandemic context preceding the beginning of our study (2020-2021) included large-scale employment terminations, furloughs, or lay-offs as businesses closed or reduced operations. Essential workers in most regions of the country were required to meet workplace masking and distancing requirements. After COVID-19 vaccines became available in the U.S. in early-to-mid 2021, many employers required vaccination as a condition of employment, particularly in healthcare, education, transportation, and customer-facing businesses. The U.S. Department of Labor (25) recommended multiple layers of protections including mask wearing, distancing, increased ventilation, and removal from the workplace of all people experiencing COVID symptoms or those having close contact with a COVID-19-infected individual (26). Also in place were more generous and lengthier unemployment benefits in the form of weekly supplements to existing amounts, expansion of eligibility to include self-employed, gig workers, and others not previously eligible for unemployment insurance, and extension of the time period people were eligible for cash benefits (27).

Given the foregoing, the purpose of this study was to understand how a group of young adults with mental health disabilities viewed the transitions they had made prior to the pandemic, and their understanding of how the pandemic had affected already-completed transitions, caused other transitions to be put on hold, and motivated the launching of new transitions. Through qualitative analysis, we sought to understand how this period of change in major life roles reflected young people’s challenges and the resilience they brought to bear on these challenges.

## Methods

### Participants

Data come from 20 respondents who met the following study inclusion criteria: 1) age 18–25 years, 2) U.S. resident, 3) diagnosis prior to the pandemic by a physician or other medical provider of one or more DSM-5 psychiatric conditions; and 4) self-reported impairment/disability specifically resulting from a mental health disorder. Exclusion criteria were: 1) cognitive impairment preventing informed consent, and 2) inability to understand spoken English.

### Procedures

Respondents were a convenience sample recruited from across the United States via web announcements posted by various youth-focused organizations and mental health advocacy groups. Interested participants were screened from January 2022, through May 2022. Of the 29 participants screened, 5 did not meet one or more eligibility criteria, 4 withdrew from consideration, and the remaining 20 enrolled in the study. All respondents provided written informed consent for study participation using procedures approved by the University of Illinois Chicago Institutional Review Board in accordance with the Declaration of Helsinki (approval # 2021-0126).

The interviews were conducted via Zoom from January 25, 2022, through March 17, 2023, by trained interviewers of the same approximate ages as respondents including young people with mental health challenges and disabilities. The initial draft of the interview protocol was reviewed by two experts on transition age young adults with mental health disabilities. Next, the 5 interviewers piloted the items with each other, and revisions were made based on their feedback to arrive at the final version. Recordings were transcribed by research staff using the Nvivo/Lumivero transcription service and then checked for accuracy by a separate researcher against transcripts generated by Zoom’s transcription function which uses Automatic Speech Recognition to convert spoken audio into text. Participants received a $40 Amazon e-card for each completed interview. Baseline interviews averaged 55 minutes, ranging from a high of 90 minutes to a low of 25 minutes. Respondent retention was 100%.

### Disclosure of interest

The authors report that they have no competing interests to declare.

### Interview schedule

The interview schedule is included in a supplement to this article. Open-ended items were designed with input from young adult research team members including those with disabilities and lived experience. Items asked respondents to describe their lives prior to the onset of widespread COVID-19 infection in the U.S., followed by changes during the initial years afterwards that they viewed as resulting from the pandemic. They also were asked about completed and in-process life course transitions both before and after pandemic onset, transitions they had expected to have completed at this stage of their lives, how their health and sense of well-being had been affected, and their self-perceived needs and motivation to address these needs. While a longitudinal qualitative design was used for the larger study, the current analysis was cross-sectional because data were limited to the baseline interviews, given our interest in transition completion prior to the pandemic and transition interruption during the pandemic’s initial stages. Subsequent publications will report on the study’s longitudinal analysis. Interviewers administered a separate demographic survey at the assessment time point immediately preceding respondents’ final interviews.

Peer researchers received the same training as other research staff which included a 2-day interviewer training that covered the study purpose and research questions, eligibility determination and recruitment procedures, interviewing skills, and observation of practice interviews followed by supervisor feedback, along with completion of university-mandated formal human subjects training. After each study visit, interviewers completed a Post-interview Self-Reflection Form (included in the supplement) that asked them to describe the interview climate, impact of the interviewer’s assumptions about the respondent on how the interview was conducted, and ways the interviewer’s personal values or beliefs shaped the probes used, interjections, and listening skills. This form was then reviewed during a one-on-one debriefing meeting with a supervisor, and a second debriefing meeting with the entire team to identify and manage bias, discuss and resolve interviewing difficulties, and develop a better understanding of cultural differences and nuances.

### Reflexivity statement

The authors have extensive experience developing and testing recovery-oriented services for people with serious mental illnesses across the lifespan. Because of this background, they recognized a tendency to view respondents through a service delivery lens and to interpret participants’ experiences using a strengths-based, recovery-oriented framework. Throughout data collection and analysis, the research team maintained reflexive analytical memos and discussed alternative interpretations to minimize the influence of individual beliefs and assumptions (28). Regularly scheduled team meetings enabled a continuous process of interrogating, examining, accepting, and articulating team members’ attitudes, perspectives, and roles (29).

### Data analysis

Univariate descriptive statistics were calculated for demographic and clinical data including means, standard deviations, and ranges for age and frequencies and percentages for categorical data using IBM-SPSS Statistics Version 31. Our qualitative analytic approach iteratively combined two complementary strategies: summative content analysis (30) and inductive thematic analysis (31). Summative content analysis allowed us to systematically apply predefined codes regarding different types of transitions commonly discussed in the literature (the deductive component) while also quantifying their occurrence at pre- and during-pandemic stages (the summative component) (32). Concurrently, thematic analysis using the constant comparative method (33, 34) was conducted to identify patterns and associated themes in young adults’ descriptions of how the pandemic impacted their transition experiences. Initial coding of baseline interview transcripts for each of the 7 life transitions was conducted by FA, BB, MB, CC, JJ, AJ, and DR, classifying transitions as: 1) completed at baseline (i.e., respondents reported that the transition had occurred prior to the initial interview); 2) in process at baseline (i.e., respondents described efforts currently being made to accomplish the transition); 3) not in process at baseline (i.e., respondents reported that they were not engaged in the transition); and 4) affected by the pandemic and its aftermath at any time (i.e., respondents specifically characterized the transition as being affected by the pandemic). Following Saldaña’s recommendations for creating analytical memos (2021), coding differences were documented and discussed, consensus was achieved and recorded, and the team created “baseline pictures” for each respondent, summarizing their transition history before and during the pandemic. Involvement of young adult co-researchers at this and subsequent stages helped ensure relevance and validity of the analysis by enabling the valuable combination of both lived experience and academic knowledge. Next, FA, MB, JC, and JJ reviewed the coding and supporting content to resolve discrepancies and identify missing information. After that, FA, MB, JC, and JJ conducted constant comparative analysis of the baseline pictures to discern patterns and associated themes that characterized participants’ descriptions of how the pandemic influenced their transitions prior to the first interview. These were shared with the larger research team for feedback and refinement, after which FA, MB, and JC met to clarify thematic definitions, settling upon a final number. Next FA, MB, and JC selected quotations from the interviews illustrating each theme, comprising the last stage of the analysis.

## Results

Demographic and Clinical Characteristics. One of the 20 respondents did not complete the demographic survey so the N for this component is 19. As shown in **Table 1**, the average age was 22.3 years (*SD* = 2.50) ranging from 18 to 25 years. Respondents could report more than one gender and detail regarding gender identification appears in **Table 1**; around half (52.6%) identified as female, 15.8% as male, and the remainder (31.6%) as gender nonbinary or other gender including transgender, genderqueer, gender non-conforming, and gender fluid. Two-thirds (68.4%) identified as White, 15.8% as mixed race or other, 10.5% as Asian, and 5.3% as Black/African American. A fifth (21.1%) identified as Hispanic/Latino. Over half had a bachelor’s degree or higher (52.6%), and around a third (36.8%) were currently enrolled in classes. Over half were employed (57.9%) and just over half (52.6%) reported total household annual incomes in the $60,000 to $89,999 range. Psychiatric multi-morbidity was common, with respondents reporting an average of 2.8 psychiatric diagnoses (*SD* = 1.4). The most frequently reported were anxiety disorders by 94.7%, depressive disorders by 63.2%, eating disorders by 42.1%, bipolar disorders by 31.6%, and PTSD by 26.3%, with lower proportions reporting diagnoses such as conduct disorder, borderline personality disorder, and dissociative disorder. Indicators of impairment due to their mental health condition included 80.0% who said they were unable to work or attend school regularly due to mental health challenges, 50.0% who had been psychiatrically hospitalized, 40.0% who had been treated at a residential facility, 35.0% who had been in special education classes and/or had an Individual Education Plan, 15.0% who reported receiving disability benefits such as supplemental security income (SSI) or social security disability income (SSDI), and 65.0% who had received accommodations from a college or university Student Disability Services office.

**Table 1:** Characteristics of young adults with disability due to mental health conditions (N = 19).

Demographic	*M*	*SD*
Age in years (range 18-25)	22.3	2.5
	*n*	%
Race		
White	13	68.4
Black	1	5.3
Asian	2	10.5
Mixed race or other	3	15.8
Ethnicity		
Non-Hispanic/Latinx	15	78.9
Hispanic/Latinx	4	21.1
Gender		
Male	3	15.8
Female	10	52.6
Genderqueer	1	5.3
Gender non-conforming	1	5.3
Gender fluid	1	5.3
Gender nonbinary	3	15.8
Transgender	2	10.5
Education		
Some high school	1	5.3
Graduated high school	1	5.3
Some college	7	36.8
BA/BS or higher	10	52.6
Income		
$0-$29,999	2	10.5
$30k-$59,999	7	36.8
$60k-$89,999	10	52.6
$90k or higher	0	0
Employed	11	57.9
Enrolled in school	7	36.8
Married or in relationship	10	52.6
DSM 5 Diagnosis		
Anxiety disorder	18	94.7
Depressive disorder	12	63.2
Bipolar disorder	6	31.6
PTSD	5	26.3
Eating disorder	8	42.1
Conduct disorder	1	5.3
Other (borderline personality, dissociative disorder)	2	10.6
	*M*	*SD*
Number of mental health conditions (range 1-7)	2.8	1.4

### Summative content analysis: transition status at baseline

Despite living with disabling mental health conditions, many respondents described their emerging adulthoods in ways fairly typical of U.S. youth, including completion of a sizable number of normative life transitions. **Table 2** presents a summary of respondents’ reported transition completions at the time of the initial interview. All but one (95%) had graduated from high school, and some had obtained undergraduate degrees or post-secondary vocational certificates. Over half (60%) had worked full-time in a career-related job or held multiple jobs that added up to full-time equivalent or more, as in the cases below.

**Table 2:** Young adults with mental health disability: transitions to adulthood completed, in process, not in process, and impacted by COVID-19 pandemic (N = 20).

Transition Status								
Transition Type	Completed at baseline		In process at baseline		Not in process		Impacted by pandemic	
	n	%	n	%	n	%	n	%
Education completion	19^a^	95	14^a^	70	--	--	20	100
Full-time or career employment	12	60	4	20	8	40	12	60
Married or intimate partner	11	55	3	15	7	35	6	30
Residential independence	15	75	2	10	3	15	6	30
Desired social support network	15	75	7	35	3	15	17	85
Identity separate from family	18	90	1	5	1	5	2	10
Parenthood	2	10	--	--	18	90	4	20

^a^ High school or post-secondary (1 respondent had not completed high school at baseline interview).

“I work for an online university in the higher education industry. I’ve been working there almost three years … working a full-time job.” (#1003, Asian, male, age 24)

“I’m a freelance tutor. [I have a] work study job through my college … I am a teaching assistant for one of the classes at the college. And I’m also a graduate assistant for one of the professors … [and] I babysit on the side. Just anything to make money.” (#1002, multiracial, female, age 23)

Over half (55%) had married or had established at least one romantic partner relationship, although some of those had ended by the time of the baseline interview. One respondent explained,

“I’m polyamorous and I have one like, I don’t know. I guess, like steady partner (laughs) for a lack of another term, and also … date other people.” (#1007, white, gender non-binary/transgender, age 23)

The number who had married was much smaller (n=3). One person explained,

“I have known [name] since I was 14 years old. We grew up as best friends and started dating after high school … I think we were only dating for a couple months until she asked me to marry her, and that was that.” (#1013, white, gender fluid, age 24)

Three-quarters (75%) of respondents had achieved residential independence, living outside the parental home, and 90% had individuated from parents, describing lives and identities separate from their families. Typically, residential independence and establishment of a separate identity occurred together, as in the following cases.

“Most of my family lives in Connecticut and New York. So, like, four-hour drive … But I really only go back to visit during the holidays, and none of them have ever been up to visit me.” (#1006, white, gender nonconforming/gender queer, age 20)

“We visit my family out in the east at least twice a year … and we also keep in touch on social media like video calls and all of that, like weekly.” (#1003, Asian, male, age 24)

Three-quarters (75%) had established a social network prior to the baseline interview, consisting of people they felt close to and considered friends. One respondent explained,

“I have a whole bunch of friends, that I’ve had pretty much had my entire life. And we keep in pretty close contact, usually over the phone.” (#1013, white, gender fluid, age 24)

Finally, only 2 respondents (10%) reported that they had become parents prior to the baseline interview. One noted,

“So now we have our daughter … She’s 4.” (#1013, white, gender fluid, age 24)

and another reported having,

“Three [children] total … they’re all three years apart … [age] six, three and two months.” (#1008, white, female, age 24)

### Transitions in process

Despite having completed many transitions, almost all respondents reported one or more transitions that were “in process” at baseline. As shown in **Table 2**, most commonly this involved completing their education (70%), typically finishing an undergraduate or graduate degree although one was completing high school. A respondent explained,

“I transferred last year, so this school is still pretty new to me … I double major in psychology and theater because I want to open up a theater therapy nonprofit for kids with autism.” (#1001, multiracial, female, age 21)

Another noted,

“I already have a bachelor’s degree … but my [trade] union would pay for me to get another associate degree, and so I just started that last year.” (#1013, white, gender fluid, age 24)

Other education transitions in process included completing certification exams or acquiring qualifications necessary to use their degree. One respondent was accumulating supervised hours toward her Licensed Clinical Social Worker (LCSW) status (#1018, white, female, age 25). Another was enrolled in a course preparing her to take the bar exam (#1011, multiracial, female, age 25).

Around a third (35%) described adding to or rebuilding their friendship networks. Some took deliberate steps to find new friends as did one who noted,

“Going to school helped in that arena, especially joining a club … most of my friends are through that club.” (#1020, white, gender non-binary, age 25)

Another described how her mental health both helped and hurt her efforts to maintain friendships, explaining,

“When I’m depressed, I can’t put as much effort into our friendship, so that makes them like kind of distant from me and me not really have many friends. But then when I’m more manic, like, I actually put more effort in because I can, and then we get closer.” (#1004, white, gender nonbinary/transgender, age 18)

Other transitions were in process among a smaller number of participants such as searching for a full-time job in their chosen career (20%), finding a romantic partner (15%), achieving residential independence (10%), or establishing an independent identity from family (5%).

### Transitions not in process

Respondents reported not actively engaging in certain transitions. This was most common for parenthood, with 90% claiming this was not a current goal. Some defined this as temporary as did one respondent who noted,

“I like to think of myself as still pretty young for having children.” (#1003, Asian, male, age 24)

However, a considerable number said they wished to remain childless, as in the following examples.

“[Parenthood] I have no plans for that ever [laughs].” (#1011, multiracial, female, age 25)

“I have no plans to have kids. [Laughs] … I like kids, but I very much cherish quiet time, and like having money.” (#1012, black, female, age 20)

“I never want kids, don’t want kids. Not having kids.” (#1014, white, female, age 25)

A smaller but still notable proportion (35%) said they were not seeking spouses or intimate partners, as did one who noted,

“I’m loving the single life.” (#1014, white, female, age 25)

and another who stopped using dating apps after deciding,

“I’m just going to focus on cultivating myself and focusing on my friendships and my family.” (#1015, white, genderqueer, age 25)

Some explained they would be open to a partner but weren’t making an effort to find one, as did a respondent who noted,

“Not actively dating. I’d be like open I guess, like if something happens, it happens. But [I’m] not looking or seeking.” (#1006, white, gender nonconforming/genderqueer, age 20)

A final transition not actively pursued by a noteworthy proportion (40%) was seeking full-time employment in the field of one’s chosen career or even working at all for those in especially poor mental health. One respondent described her decision to prioritize completing a college degree over working

“…because I can’t do both anymore.” (#1014, white, female, age 25)

Others noted that their mental health interfered with pursuing full time work,

“I expected myself to be … good enough mentally to be able to keep a job, but I don’t think I am.” (#1004, white, gender nonbinary/transgender, age 18)

Another felt she needed to complete her therapeutic day program first, and explained,

“I’m just doing some more casual [work] until day hospital is finished and I feel like I can hold down … a 9 to 5 office job. I want to make sure I’m truly ready.” (#1017, white, female, age 24)

Finally, some had chosen to forego employment as did one respondent who noted,

“I’m trying to get [on] disability [benefits].” (#1005, white, female, age 19)

Others found that unemployment benefits were an attractive alternative to searching for limited career-oriented job opportunities during the pandemic. One woman was called back to work after being laid off due to the pandemic but explained,

“I made so much more money on the pandemic unemployment than I did working. I was like, ‘I’m not going to go back to work’.” (#1014, white, female, age 25)

Other more infrequent transitions not in process were residential independence (15%), building their friendship network (15%), and individuating from family (5%).

As shown in the far-right hand column of **Table 2**, every respondent (100%) reported one or more effects of the pandemic on their transition experiences. All respondents (100%) reported pandemic-related changes to educational pursuits, usually negative. A large majority (85%) described ways COVID affected their friendship networks, usually but not uniformly negatively. Over half (60%) described negative impacts on obtaining full-time employment in their chosen fields. Lower percentages cited either positive or negative pandemic effects on their spousal or intimate relationships (30%), residential independence (30%), parenthood (20%), and individuation from their families (10%).

### Thematic analysis: transition interruptions attributed to the COVID pandemic

**Table 3** presents 6 categories that arose from a thematic analysis of transition changes respondents attributed to the pandemic: 1) pandemic-related educational changes had effects that extended beyond academic learning; 2) friendship networks were altered due to pandemic restrictions; 3) current life situations were not what they expected because of the pandemic; 4) pandemic changes led them to reevaluate and alter their career paths; 5) the pandemic job market discouraged them from seeking full-time work or, in some cases, any work at all; and 6) transitions accelerated by the pandemic led to earlier than expected independence and maturity.

**Table 3:** Thematic analysis of transition disruptions due to the COVID-19 pandemic among young adults with mental health disability (N=20).

Theme number & description	# participants	Example quote	Sub themes
1. Pandemic-related educational changes had effects that extended beyond learning	9	“Before the pandemic … I didn’t have to buy a lot of food because [name of department] provided food all the time … if there was an event and they didn’t eat all the food they just leave it [out] … Or you’d crash an event and get food there. Now they don’t for obviously, safety precaution reasons.”	a. residential challenges b. food insecurity c. lack of access to reasonable accommodations d. scholarship access issues e. loss of income
2. Social networks were affected by pandemic	15	“If anything it’s enriched my relationship with my friends. And I’ve made more friends as a result … of the pandemic.”	a. positive effects on friendships and social circle b. contacts dropped off due to pandemic restrictions c. differential effect depending on pre-pandemic situation
3. Current life situation not what they anticipated at this point in their lives due to the pandemic	6	“I figured I would [be] teaching full-time. I had expected to still be able to keep up with horseback riding, and … probably by now I would have started my master’s degree … And I would have my mental health figured out and I’d be able to … live without it impacting me as much.”	a. not adhering to societal timeline for transition completion b. not achieving specific life goals by original timeline
4. Career path was altered due to the pandemic	4	“[I realized] I didn’t like what I was doing with business … honestly, the pandemic kind of helped me make my transition from business to psych … I feel like I had a lot more free time to think. (laughs)…I realized I had to do something that I liked.”	a. realized they didn’t like current career path b. unable to complete career path step so changed career goal
5. Transitions accelerated by the pandemic led to earlier independence and maturity	5	“I think COVID forced me to kind of take responsibility for my life in a way that I hadn’t before and like, that’s changed my entire worldview.”	a. already ongoing transitions were accelerated b. new transitions started or completed earlier than anticipated
6. Pandemic led them to stop looking for or holding fulltime work in chosen field	7	“I made so much more money on the pandemic unemployment than I did working. I was like, ‘I’m not going to go back to work’.”	a. realized not ready for fulltime work in chosen field due to poor mental health b. discouraged by lean job market c. realized financial benefit of receiving unemployment or disability benefits

Young Adults with Mental health Disability (N = 20).

### Pandemic-related educational changes had effects that extended beyond academic learning

Pandemic-related changes in schooling included classes and internships becoming remote or being cancelled, decreased or more impersonal attention from teachers, needs for new or better technology, and withdrawal of other education-related supports. Thematic analysis revealed that these changes created challenges extending beyond learning itself. One of these was residential challenges, which sometimes turned from negative to positive. For example, one respondent described a negative/positive experience as follows:

“…[we could] no longer stay in the [dorm] rooms … That was super alarming to me, but turned out all right because I got to room with [name] … And while we were friends before COVID I don’t think we’d be as close of friends if we had not lived together.” (#1006, white, gender nonconforming/genderqueer, age 20)

But other times, losing their residence and being forced to move home caused significant strain and threatened independent identities. As one woman who returned to live with parents during the pandemic explained,

“I’m 24 but I still live at home … my parents kind of still treat me like a child (laughs) so I try to stay [at boyfriend’s apartment] as much as I can just to have some sort of freedom. But I’m a grad student, I work full time, I pay for my tuition myself.” (#1002, multiracial, female, age 23)

Other ramifications of the pandemic’s impact on education transitions included food insecurity, mentioned by several respondents. As one noted,

“Before the pandemic … I didn’t have to buy a lot of food because [name of department] provided food all the time … if there was an event and they didn’t eat all the food they just leave it [out] … Or you’d crash an event and get food there. Now they don’t for obviously, safety precaution reasons.” (#1011, multiracial, female, age 25)

Another reported relief at being accepted into

“…a scholarship to eat food at the university. So I’ve been eating a lot more healthier.” (#1016, white, male, age 19)

Another ripple effect from pandemic-related education changes was economic. This could hit work-study students especially hard, even when their academic institutions tried to help. As one explained,

“[The university said] we will pay you the rest of what we think you would have earned had you been truly working … They did give me money … but they did not give me what would have been the full earnings … even though that’s what they said they were going to do.” (#1006, white, gender nonconforming/genderqueer, age 20)

Others who depended on financial aid were forced into unwanted heavy online course loads, as in the case of one respondent who explained,

“In order to get my financial aid, I had to be full time. So, I was in like five courses at once.” (#1008, white, female, age 24)

In still another case, pandemic-related reductions in financial aid forced a respondent to switch from attending a more prestigious school to a less expensive college closer to home (#1001, multiracial, female, age 21).

### Friendship networks were affected by the pandemic

A second area affected by the pandemic was respondents’ friendship networks. Interestingly, these effects could be positive or negative, with restrictions on social contact and infection precautions acting sometimes in unexpected ways. As one noted,

“I couldn’t see people in person and we stopped hanging out. And some of them I still talked to over text, but it’s not the same.” (#1005, white, female, age 19)

But others described how the pandemic had bolstered their networks, as did one who explained,

“If anything it’s enriched my relationship with my friends. And I’ve made more friends as a result … of the pandemic.” (#1007, white, gender non-binary/transgender, age 23)

Another respondent noted that having more free time during the pandemic allowed close friendships to become closer but diminished bonds with more distant acquaintances,

“My closest friends, I feel like we’ve actually gotten closer because … we were taking advantage of the fact that we had more flexible time and so we were … getting in better contact … My friends who were not my closest friends … communication dropped off a lot more … I wasn’t seeing them anymore.” (#1015, white, genderqueer, age 25)

### Current life situations were not what they had anticipated at this point in their lives

Some respondents expressed a sense of off-timedness (35) resulting from the pandemic. This concept from life-course research refers to feelings of depression or anxiety at not meeting culturally and socially defined developmental milestones within expected time frames (36, 37). One respondent lamented,

“I thought I would be a lot further in my college classes like further toward my degree. And I thought that I’d be on my own feet and not need help from my parents.” (#1005, white, female, age 19)

Another was unstably housed due to high apartment rental rates resulting from the pandemic and observed that,

“I never expected to be living in a minivan (laughs).” (#1007, white, gender nonbinary/transgender, age 23)

Still another had an unexpected pregnancy during the pandemic and noted,

“I thought I would be finished with school, maybe working in a career with my degree … Well, I ended up getting pregnant.” (#1008, white, female, age 24)

Others described a disconnect between their personal aspirations and current circumstances resulting in distress due to unmet expectations (38). Psychological research shows that gaps between individualized expectations young people have for their futures and lives that fail to meet these expectations are associated with poor mental health (39). One woman explained her regret as follows:

“I figured I would [be] teaching full-time. I had expected to still be able to keep up with horseback riding, and … probably by now I would have started my master’s degree … And I would have my mental health figured out and I’d be able to … live without it impacting me as much.” (#1017, white, female, age 24)

Some were unable to realize plans to start their own businesses, as did one respondent who explained,

“I wanted to continue my education and get a Masters [degree] in automotive engineering … I wanted to get a big space, then set up my shop, but … the prices went up for rent.” (#1019, white, male, age 23)

Yet another respondent was forced to forego plans to adopt a child, noting,

“My spouse and I had been going through the process to become adoptive parents. But when COVID hit we put that on the back burner … we couldn’t go to UPS and get our fingerprints done, we couldn’t … do all of the things … to become qualified.” (#1013, white, gender fluid, age 24)

### Career paths were altered due to the pandemic

Pandemic-related changes led some respondents to reconsider and revise their career paths. One respondent described this process:

“I was a business major and the pandemic canceled my internship … so I started working as a caregiver for a child with autism … [I realized] I didn’t like what I was doing with business … honestly, the pandemic kind of helped me make my transition from business to psych … I feel like I had a lot more free time to think. (laughs)…I realized I had to do something that I liked.” (#1002, multiracial, female, age 23)

Another explained,

“I had just completed the hours that I needed of supervision, and submitted all my paperwork, and was approved to take … my [teaching] certification exam in the end of March of 2020, and they canceled it … And so, by the time the testing center said [I could] reschedule, I was like ‘I don’t even wanna do this anymore’…[later switched to social work major].” (#1020, white, gender nonbinary, age 25)

### Accelerated transitions due to the pandemic led to early independence and maturity

For some, the pandemic sped up existing transitions or prompted new transitions earlier than expected, leading to a newly found sense of independence and maturity. As one participant who had been in an abusive relationship at the start of the pandemic explained,

“I think COVID forced me to kind of take responsibility for my life in a way that I hadn’t before and like, that’s changed my entire worldview. (#1007, white, gender nonbinary/transgender, age 23)

Housing changes were often implicated in early maturity. One young man who was living with his mother prior to the pandemic noted,

“I guess the thing that I was most proud of is still being able to get a job and move out during the pandemic … despite everybody losing their job, the amount of people on unemployment … I felt very proud of being able to achieve that.” (#1016, white, male, age 19)

Losing university housing accelerated residential independence for others, as in the case of a respondent who had to leave her university’s dorm:

“If it weren’t for COVID I probably would have done another year in the dorms … I ended up moving in with my boyfriend at the time, and it was my first apartment, my first adult [responsibility] paying bills, and … living on my own … and I will say that I learned a lot.” (#1012, black, female, age 20)”

### Pandemic challenges led some to stop looking for or working at full-time jobs in their chosen fields

The poor pandemic job market discouraged some participants from seeking full-time jobs in their chosen professions. Others found that the pandemic had drastically altered their jobs in ways that negatively affected their mental health and reduced their desire to continue in their chosen field. A teacher who had been working full-time recounted “So the kindergarteners were not a big fan of the masks and it was very hard enforcing that. The area that that school was in had very strong opinions about Covid and so a lot of the kids said,

“Well, my dad said I don’t have to wear a mask,” and so that was … difficult because I had to enforce you know state and school policies … For my own mental health … I think that I’m not going to go into a classroom and teach for at least 2 years.” (#1017, white, female, age 24)

Some participants felt their poor mental health was not conducive to full-time work as did one who noted,

“I decided that … I couldn’t like mentally work and do school at the same time anymore … I don’t think I’m going to work until I get out of school because I can’t do both anymore.” (#1014, white, female, age 25)

Another participant was taking college courses and had “…a lot of therapy appointments” so decided to forgo employment and apply for disability benefits instead (#1005, white, female, age 19).

## Discussion

Study results suggest that this group of young people who had experienced disability related to mental health conditions had nevertheless completed a sizable number of transitions to adulthood prior to the pandemic. All but one had completed high school and over three-quarters had become residentially independent, had established identities separate from their families, and had formed what they considered to be satisfying social networks. These young adults appear to have negotiated emerging adulthood much like their non-disabled counterparts and exhibited a largely normative life trajectory at study baseline, despite experiencing mental health-related disability.

Since over 70% were engaged in post-secondary education, career-building activities, or vocational training at the time of pandemic onset, it is not surprising that this was one of the major areas impacted. Respondents described challenges with the switch to remote instruction including difficulty learning, lacking appropriate technology, and inability to relate satisfactorily to both teachers and other students. These educational changes had ripple effects beyond the classroom that included residential instability, uncertainty about financial aid, food insecurity, and work-study job fluctuations. Previous research has noted how economic and emotional vulnerabilities intersected and interacted with inequalities of mental health, well-being, and learning among college students during the pandemic (40). This includes the deleterious effects of losing in-person instruction, not only because it promotes strong engagement between students and teachers, and students and their peers, but also because it provides direct access to the full-range of academic and wraparound services that a school provides (41). Indications are that these negative effects have been disproportionately experienced by students with disabilities (42), and this was evident in the lives of our respondents.

Another major impact was on transitions to full-time or full-time equivalent employment, especially jobs in the respondents’ chosen fields. Interestingly, over half of the young adults we interviewed had achieved this transition prior to baseline. However, many of these jobs had ended involuntarily during the pandemic. Other career-related job endings were directly connected to the pandemic, such as the respondent in a full-time teaching position who quit because her students wouldn’t wear masks, and other respondents who decided not to return to the jobs they had held after being laid off and called back to work. Struggling to balance the risks of COVID exposure with the need for employment was noted in another study of low-income first year college students with a history of poor mental health (40). Still other respondents decided to pursue post-secondary education and training for second careers. It remains to be seen whether this group of highly motivated young people will eventually realize their career ambitions. A study conducted at a first episode psychosis clinic in January through September 2020 found that employment loss among participants was significantly greater and lasted significantly longer compared to the period prior to the COVID pandemic (43).

A large proportion of respondents had decided they did not want to make the transition to parenthood either currently or, for some, ever in their lifetimes. This is reflected in the larger U.S. culture’s trend toward delayed childbearing in a world undergoing rapid social, economic, and technological transformations (44). This trend is now in evidence globally as well (45). A recent U.S. Census Bureau working paper (46) found that young adults age 25–34 are prioritizing financial stability such as steady employment and independent living over traditional milestones including parenthood, due to increased costs of living, student debt, and housing challenges. Our respondents’ feelings about parenthood may be part of this larger societal and international trend.

Study findings contradict the assumption that young people with significant mental health challenges lack the motivation and resilience needed to navigate complex life trajectories during a global pandemic (47, 48). While they surely faced both internal and external obstacles, their accounts of their experiences also demonstrate resourcefulness and a sense of optimism about their futures. Others have noted that people with disabilities, while surely dealing with multiple challenges related to health vulnerabilities and unfavorable social determinants (49) also coped successfully with the pandemic’s effects by drawing upon personal expertise and external support grounded in interconnectedness and mutual aid (50). This is echoed by our respondents’ descriptions of emotional support and practical assistance from family and friendship networks that were in place pre-COVID and were supplemented or in some cases re-built following pandemic onset. Moreover, regarding resilience, a study of distress levels during the pandemic among U.S. young adults who self-reported pre-existing mental health conditions (15) found that those with high levels of mental health symptoms plus large numbers of pandemic-related impacts exhibited significantly *higher* resilience levels than their low-symptom/low COVID-impact counterparts. The authors noted that these youths may have been better equipped to handle severe stress and adversity thanks to skills and resources they possessed and were experienced in using. This is reflected in our study’s findings that respondents described unexpected and newfound resourcefulness, independence, and maturity.

Finally, our results illustrate the extent to which respondents compare their life transitions to not only societal expectations regarding developmental milestones but also their personal aspirations for the kinds of lives they want to live. This was reflected in disappointment and regret expressed by those still in school or unemployed or residing with parents when they had expected to achieve the opposite statuses. It was also displayed by those who lamented foregone plans such as starting a business or adopting a child or earning an advanced degree. Emotional distress resulting from both types of disappointment is well documented in this age group (51) and especially so when plans can be characterized as “ambitious but achievable” versus “unrealistic or completely out of reach” (38). Prior research on young adults with mental illness underscores their need to redefine themselves by achieving valued life roles that enhance social inclusion and acceptance (52). The fact that some young people had aimed high meant that they were forced to accommodate transition failures, although the pandemic context could cushion feelings of disappointment.

These findings underscore the need for transition-focused wraparound service systems that sustain young adults with mental health disabilities through periods of societal upheaval—addressing housing, employment, financial security, support for continuing education, and peer connection alongside clinical needs (53, 54). High levels of psychiatric comorbidity reported by our participants support use of a transdiagnostic and dimensional approach and early intervention strategies to avoid development of further comorbidities (55). Also important is guiding young people to services and supports that scaffold their complex emerging identities and build on evident strengths rather than destabilize and alienate them by emphasizing compliance and conformity to service system demands (56). Another critical component is incorporating informal support networks such as friends and family members identified as helpful by our participants and shown to be pivotal to young adult behavioral health help-seeking and recovery (57).

### Study limitations and strengths

One strength of our study is the rich data describing respondents’ experiences in their own words from their individual perspectives. Another is the involvement of young people with lived experience of mental health challenges and other disabilities as interviewers, data analysts, and co-authors. Their rapport with respondents and shared understandings helped to surface alternative interpretations, clarify ambiguous meanings, and identify interconnected influences that might have remained obscured (56). A weakness is that no causal inferences can be drawn from the study’s results. In addition, data in this analysis were cross-sectional rather than longitudinal, which may have influenced study findings given changes in respondents’ perspectives over time. The transitions explored in this research are culturally bound and their nature and timing may not be relevant in all countries or viewed similarly by subgroups of individuals in those countries (58, 59). Finally, there were specific groups of respondents who were underrepresented in our convenience sample such as people with schizophrenia and respondents from the west and southwestern U.S.

## Conclusions

This study provides a qualitative portrait of young adults with disabling mental health conditions navigating the transition to adulthood before and during the COVID-19 pandemic. Despite significant psychiatric multi-morbidity and pandemic-related disruptions, participants demonstrated a largely normative pre-pandemic life trajectory and showed notable resilience in the face of extraordinary adversity—challenging deficit-centered narratives about this population. Participants drew on social support networks, adapted their goals, and maintained optimism about their futures. Their views on parenthood similarly reflected informed agency consistent with broader generational trends, rather than impairment-driven avoidance.

Future research should employ larger, more representative samples and longitudinal designs in both the U.S. and internationally, to examine how intersecting factors such as country of residence, socioeconomic status, race/ethnicity, and gender identity shape transition outcomes over time. Such studies will benefit substantially from inclusion on the research team of young adults with lived experience of mental health challenges and other disabilities, whose perspectives make invaluable contributions at all stages of the research process.

## Data Availability

At study completion, deidentified data will be added to an existing data bank at the Interuniversity Consortium for Political and Social Research (https://www.icpsr.umich.edu/sites/icpsr/about) located at the University of Michigan as required by U.S. law.
