# Supplementary material for: Transitions to adulthood among U.S. young adults with mental health disabilities before and during the COVID-19 pandemic: traveling multiple pathways in uncertain times
Source: Front Psychiatry. 2026 Sep 11;17:1911182. doi: 10.3389/fpsyt.2026.1911182 (PMC13613812; doi:10.3389/fpsyt.2026.1911182)
Supplement: Supplementary file 1 [file SupplementaryFile1.docx]

**Transitions to Adulthood Among U.S. Young Adults with Mental Health Disabilities Before and During the COVID-19 Pandemic: Traveling Multiple Pathways in Uncertain Times**

**Article Supplement**

**Pivot Toward Wellness: Time 1 Interview Schedule**

Hi [**name of respondent**]. Thanks for joining me today for your **first [second, third, etc.]** interview of the Pivot Toward Wellness Study.

As a reminder, this study focuses on the effects of the COVID-19 pandemic on your life, and any ways it has disrupted your life and important life transitions. Please remember that your participation in this study is ***completely voluntary.*** All of the answers you give me today will be kept confidential. We will not share what you say with anyone without your permission. The only exception is if you tell me about abuse or harm to yourself or others. Then I will call your Secondary Contacts or the appropriate authorities, depending on the situation. The interview will take about 1 hour and you will receive $40 for completing it. As we go through the interview, you don’t have to answer any questions you don't want to. If you don't understand something, please ask me to explain it.

Do you have any questions? [**respond to any queries**]. Ok, let’s get started.

1. Tell me how you spend your time in a typical week.

Use these probes if R has not discussed:

- Are you in school or taking any classes?
- Where are you living and with whom?
- Do you have a job? Are you looking for work?
- What kinds of things do you like to do in your spare time?
- Do you have people you consider friends?
- Are you close with your family?
- Do you have a romantic partner?

2. How would you describe your strengths or the positive qualities you have?

3. Think back to your life at the beginning of 2020. I’m referring to January and February ***two years ago, that would be [insert number] months ago,*** before the pandemic started. Please describe a typical week for you back then.

4. Before the pandemic started, where did you think you’d be at the end ***of last year – 2021***?

**Allow R to answer first. Then Probe:**

- Did you think you’d be working, in school, living on your own, parenting, that kind of thing?

5. Let’s talk more in-depth about any ways COVID-19 may have interrupted your life. I’m going to ask about different life areas. Please tell me whether and how the pandemic has kept you from achieving what you wanted in each area. Please note that some of the questions may or may not apply to you at this moment. If this is the case let me know.

a. Let’s start with your friendships. Has COVID interrupted your relationships with friends? If so, tell me how.
b. How about your education or training for a career? Has COVID interrupted that?
c. How about having a romantic partner? Has COVID interrupted your hopes for a romantic relationship if you wanted one?
d. How about where you’re living? Has COVID interrupted your plans for where you live and who you live with?
e. How about working? Has COVID interrupted your employment or plans to get a job?
f. How about your family relationships? Has COVID interrupted how you interact with members of your family?
g. How about having children and parenting? Has the pandemic interrupted your plans in that area in any way?

Thank you for sharing that important information with me. We’ve been talking for a while. Would you like to take a 5-minute break?

6. Next, I’d like to know about your **physical health**. How is your physical health now? Are you managing any health conditions or illnesses?

7. How would you describe your current wellness routines, in terms of things like healthy eating, physical activity, sleeping, etc.?

8. Have there been any changes in your health since the pandemic began?

Probe for whether R was diagnosed with COVID-19.

9. Next, let’s talk about the pandemic’s effect on your **mental health**. When you compare your mood now to what it was like before the pandemic, are there differences you’ve noticed?

10. Were there times in the past month where you stayed home either because you’d been exposed to the virus and didn’t want to infect others, or, there was concern that others would infect you? Could you describe those times?

11. Can you describe how your vaccination status has influenced your life?

12. Let’s think about [**two months/four months**] from now, which would be [month and year]. What do you expect to be doing in the different life areas we talked about, like school, work, where you live, your family, friends and romantic relationships, and favorite hobbies or things you like to do?

13. What the thing you’re most proud of in dealing with the COVID-19 pandemic?

**Post-interview Self-Reflection Form**

**Date of Interview: ____/____/____**

**Start Time: ____:_____ ☐ AM/ ☐ PM	End Time: ____:_____ ☐ AM/ ☐ PM**

**Interviewer: ☐ CC	☐ AJ	☐ DR	☐ CS	☐ FA**

**Interview Number: ☐ 1 ☐ 2 ☐ 3 ☐ 4 ☐ 5 ☐ 6	Missed Interview: _____, _____, _____**

**General Impressions:**

1. Were you able to put the participant at ease? If not, why?
2. Did any of the questions make the participant uncomfortable when you first asked or probed for more details. If so, please explain.
3. Could the questions have been phrased differently to achieve the expected outcomes? Please explain.
4. Did anything unexpected impact the progress of the interview?
5. Was there sufficient time allowed for the interview?

**Assumptions**

6. What assumptions did you make about the participant before you started the interview?
7. How did these assumptions affect how you asked the questions, your interjections, and your listening skills?

**Values/Beliefs**

8. How did your personal values or beliefs affect or shape the probes you used, your interjections, and your listening skills?

**Connection with Participant**

9. How did your emotions or feelings about the participant affect or shape the probes you used, your interjections, and your listening skills?

**Final Impression**

10. Do you feel the way you conducted the interview will affect the analytical process in any way?
